# Subclinical Leber’s hereditary optic neuropathy with pediatric acute spinal cord onset: more than meets the eye

**DOI:** 10.1186/s12883-018-1227-9

**Published:** 2018-12-27

**Authors:** Eleonora Mauri, Robertino Dilena, Antonio Boccazzi, Dario Ronchi, Daniela Piga, Fabio Triulzi, Delia Gagliardi, Roberta Brusa, Irene Faravelli, Nereo Bresolin, Francesca Magri, Stefania Corti, Giacomo P. Comi

**Affiliations:** 1 Department of Pathophysiology and Transplantation, Dino Ferrari Centre, University of Milan, Milan, Italy; 2 Foundation IRCCS Ca’ Granda Ospedale Maggiore Policlinico, Unit of Neurophysiopathology, Milan, Italy; 3 Foundation IRCCS Ca’ Granda Ospedale Maggiore Policlinico, Pediatric Unit “Media Intensità di Cura”, Milan, Italy; 4 Foundation IRCCS Ca’ Granda Ospedale Maggiore Policlinico, Neurology Unit, Milan, Italy; 5 Foundation IRCCS Ca’ Granda Ospedale Maggiore Policlinico, Neuroradiology Unit, Milan, Italy

**Keywords:** Leber’s hereditary optic neuropathy, Spinal cord, Pediatric, Mitochondrial pathology

## Abstract

**Background:**

Leber’s hereditary optic neuropathy (LHON) is a mitochondrial disease characterized by visual loss consequent to optic nerve atrophy. In some cases, LHON is associated with heterogeneous neurological extraocular manifestations and is referred to as “Leber plus disease”; rarely it is associated with a multiple sclerosis (MS)-like syndrome known as Harding disease, but no pediatric extraocular acute spinal onset is reported.

**Case presentation:**

We describe the case of a 5-year-old girl carrying the G3460A mtDNA mutation who was referred to clinical examination for bilateral upper and lower limb weakness with no sign of optic neuropathy. Spinal cord MRI showed hyperintense signal alterations in T2-weighted and restricted diffusion in DWI sequences in the anterior portion of the cervical and dorsal spinal cord resembling a spinal cord vascular injury. No association between this mutation and pediatric spinal cord lesions has previously been reported. Alternative diagnostic hypotheses, including infective, ischemic and inflammatory disorders, were not substantiated by clinical and instrumental investigations.

**Conclusions:**

Our case reports a novel pediatric clinical manifestation associated with the m.3460G > A mtDNA mutation, broadening the clinical spectrum of this disease. Early identification of new cases and monitoring of carriers beginning in childhood is important to prevent neurological deterioration and preserve long-term function.

## Background

Leber’s hereditary optic neuropathy (LHON) is a maternally inherited genetic disease that occurs due to a mitochondrial DNA (mtDNA) mutation that causes central, bilateral, painless, progressive visual loss due to optic nerve atrophy, particularly in young adult men [1]. Three disease-causing mutations that affect subunits of complex I of the mitochondrial respiratory chain (MTND1: m.3460G > A, MTND4: m.11778G > A, and MTND6: m.14484 T > C) are responsible for 90% of the cases. The extraocular manifestations of the disease, known as “Leber plus disease”, include movement disorders, mental retardation, seizures, cerebellar ataxia, and peripheral neuropathy [1]. Other associations, such as multiple sclerosis (MS)-like syndrome, referred to as “Harding disease” or “LHON-MS” [2], Leigh-like encephalopathy and MELAS (mitochondrial encephalomyopathy, lactic acidosis and stroke-like episodes)/LHON overlap syndromes, have also been reported [3]. We describe a 5-year-old girl affected by an acute spinal cord lesion mimicking a vascular lesion. The patient had a family history of LHON due to the G3460A mtDNA mutation.

## Case presentation

A 5-year-old girl was admitted to our Emergency Department after an episode of acute interscapular back pain occurring without trauma and followed by bilateral upper and lower limb weakness.

**Fig. 1:**
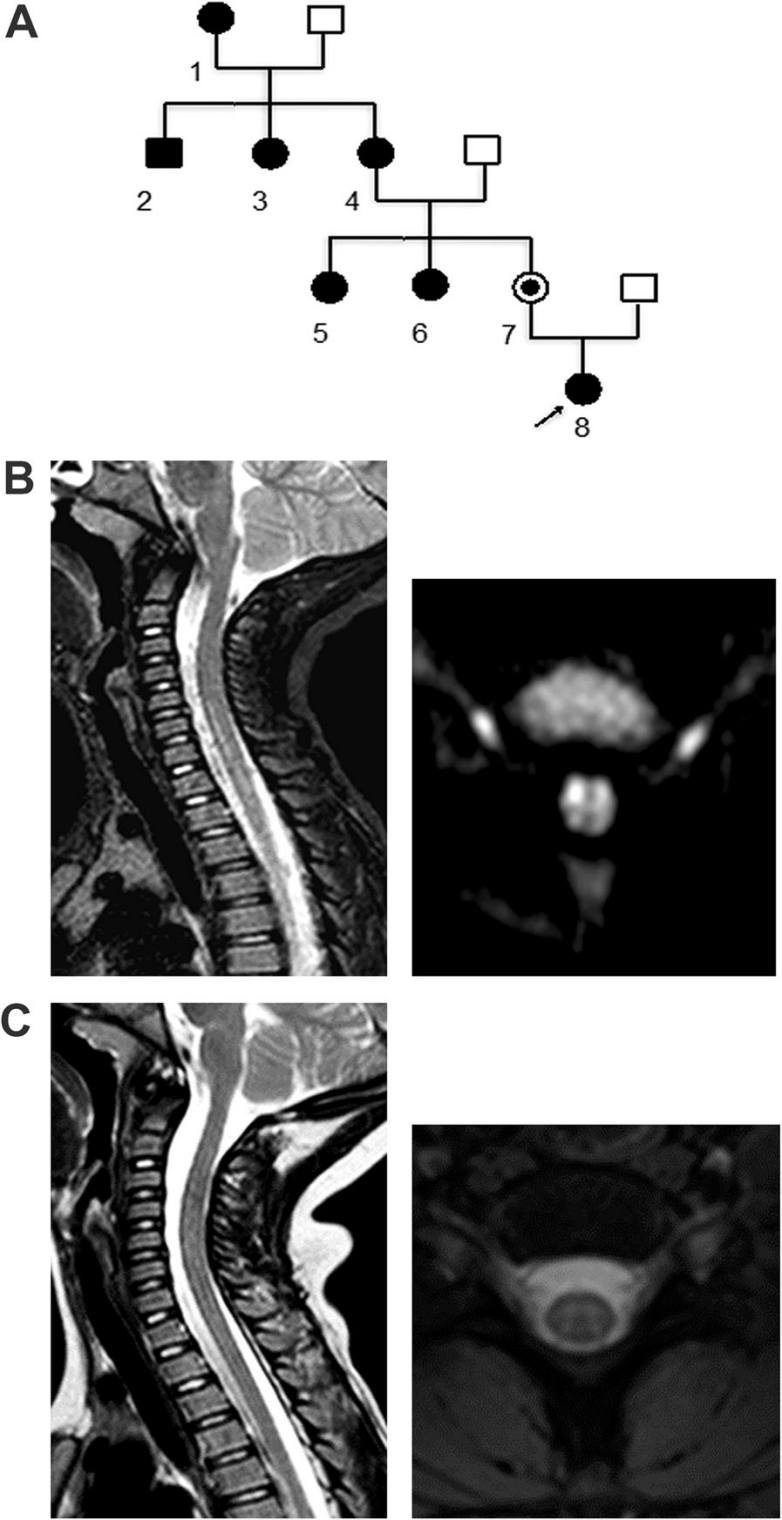
Family pedigree and MRI spinal cord imaging. **a**. Family pedigree; the patient is number 8. **b**. Spinal cord MRI T2-weighted/DWI sequences indicating a hyperintense cervical-dorsal lesion. **c**. Spinal cord MRI T2-weighted/DWI sequences obtained 10 days later, showing regression of the hyperintense signal

Her family history included 8 Italian members harboring the same homoplasmic m.3460G > A mtDNA (Table 1, Fig. 1a). All the family members presented headache poorly responsive to NSAIDs and, except for the girl and her mother, visual loss due to optic nerve pathology. The patient’s medical history was unremarkable.

**Table 1:** Clinical findings at prolonged follow-up of the entire family. No family member presented signs of a spinal cord lesion or pediatric onset of the symptoms. The individuals in the family pedigree are identified as follows: 1 Great-grandmother; 2, Uncle; 3, Uncle; 4, Grandmother; 5, Aunt; 6, Aunt; 7, Mother; 8, Proband

	1 Great grandmother	2 Uncle	3 Uncle	4 Grandmother	5 Aunt	6 Aunt	7 Mother	8 Proband
*Gender*	F	M	F	F	F	F	F	F
*Mutation G3460A*	Heteroplasmic	Homoplasmic	Homoplasmic	Homoplasmic	Homoplasmic	Homoplasmic	Heteroplasmic	Homoplasmic
*Age at onset -y*	Unknown	Unknown	Unknown	30	21	9	–	5
*Signs of onset*	Ipovisus	Ipovisus	Iipovisus	Ipovisus Unilateral to bilateral optic neuritis	Ipovisus Unilateral optic neuritis	Ipovisus Bilateral optic neuritis	No signs at ophthalmologic visit 2011	Spinal cord lesion
*MRI brain signs*	–	–	–	Multiple periventricular subcortical lesion	–	–	–	Negative
*OCB in CSF*	–	–	–	Absent	–	–	–	Absent
*Progression of the disease*	Blindness	Blindness Headache	Blindness Psychiatric comorbidities	Vertigo Tremor Diplopia Psychiatric trait	Bilateral scotoma Headache	Monolateral scotoma left eye; blind right eye	–	–
*Disorders associated*	Unknown	Unknown	Unknown	Rolandic epilepsy Pericarditis ANA pos.	Osteoid osteoma	LLAC pos. Connectivitis	Headache	Cutaneous mastocytosis
*Therapy*						Idebenon	–	Aspirin Corticosteroids Idebenon
*Follow-up duration*	20 ys	20 ys	20 ys	20 ys	7 ys	14 ys	5y	1 y

**Fig. 2:**
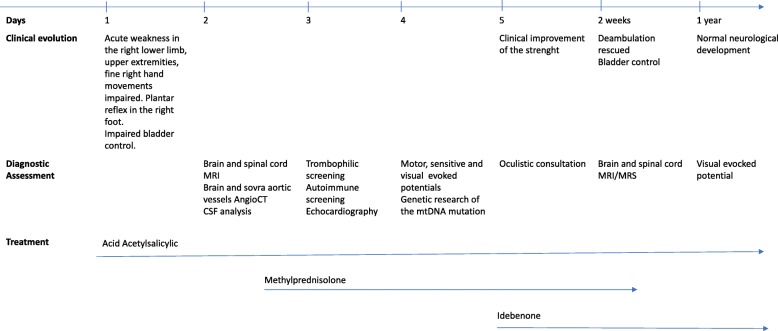
Timeline

The patient’s vital signs were normal and stable. At the neurological examination, her cognitive functions were normal. There were no cranial nerve lesions. She was unable to stand and walk and had more severe weakness in the right lower limb. She also presented weakness in her upper extremities and difficulties with fine hand movements, particularly in the right hand. The right patellar and Achilles tendon reflexes were brisk. The plantar reflex was positive in the right foot. She presented impaired bladder control. No alterations in touch or pain sensitivity were present in the trunk and limbs. No concomitant signs of infection or inflammation were present, and no such signs had been reported in the previous weeks. Analyses of CSF pressure, glucose, protein, cell count, viral PCR, and culture were normal, and oligoclonal bands were absent (see timeline of events and treatment in Fig. 2). The patient underwent a spinal cord MRI; the results showed hyperintense signal alterations in T2-weighted sequences and restricted diffusion in diffusion weighted imaging (DWI) sequences in the anterior portion of the cervical and dorsal spinal cord, suggesting anterior spinal artery territory involvement (Fig. 1b). Computed tomography angiography (CT) imaging showed no arterial dissection or other vessel abnormalities. Visual evoked potentials were normal. Somatosensory evoked potentials in both legs showed decreased conduction velocities. Motor evoked potentials showed lower amplitude for cortical derivation, prolonged latency in the upper limbs and normal in the lower limbs. Central conduction time was increased in the upper limbs and normal at the lower limbs. Complete autoimmunity and thrombophilia screening were unremarkable. Testing for anti-AQP4 antibodies was negative, and anti-MOG antibodies were not significantly elevated. A cardiologic consultation and echocardiography identified normal heart and aorta features. At the ophthalmological assessment, pupillary reactions were normal, the fundus oculi did not reveal pathological signs, and color vision was not affected. The visual acuity was 9/10 bilaterally. The digital visual field test displayed a mild defect in the peripheral portion of the visual field that was more evident in the left eye. Optical coherence tomography, visual evoked potential and electroretinograms were normal. As the child grows, her visual function will require careful monitoring, particularly when she reaches the adolescence.

After obtaining written informed consent consistent with the principles set forth in the Declaration of Helsinki, total DNA was isolated from the patient’s peripheral blood according to standard protocols. Full-length mitochondrial DNA sequencing was conducted according to a previously described protocol [4], and detected the haplogroup H27 was detected in the proband. The following variants were detected in the homoplasmic state: m.41C > T, m.73A > G, m.263A > G, m.1438A > G, m.3460G > A, m.4769A > G, m.8860A > G, m.11719G > A, m.15326A > G, m.16129G > A, m.16316A > G, m.16519 T > C. Based on the presence of the m.11719G > A and m.16316A > G nucleotide variants, the patient is predicted to belong to the H27 haplogroup. The m.3460G > A transition, a major mutation associated with LHON, was verified in blood-derived DNA of other family members by PCR-RFLP analysis.

Acetylsalicylic acid was administered at low doses (2.5 mg/kg daily), and high doses of methylprednisolone (20 mg/kg daily) were empirically administered for three days. Although no proven treatments for LHON are available, early treatment with idebenone is thought to limit the progression of the disease; the patient was administered 45 mg of idebenone three times daily. Within two days following this therapy, clinical manifestations improved, and the patient regained bladder control and the ability to ambulate; within one week, the girl also recovered nearly normal strength in both arms. A control spinal cord MRI together with 1H-MRS to study the lactate peak was performed 10 days after hospital admission; the results showed complete regression of the alterations and no abnormal metabolites (Fig. 1c). The patient continued outpatient rehabilitation, and her motor functions improved, resulting in an almost completely normalized neurological examination after 2 months and preserving these achievements at follow-up one year later.

## Discussion

In the case described here, the acute onset associated with back pain and the spinal cord MRI alteration in the region of the anterior spinal cord artery could first suggest an arterial infarction. Indeed, LHON can be associated with cardiac arrhythmias and can predispose individuals to embolic events [5]. The patient’s symptoms improved rapidly after corticosteroid treatment, and there was a significant reduction of the signal in the spinal cord MRI ten days later, a pattern more consistent with an inflammatory origin of the lesions. A condition characterized by MS-like neuropathological and clinical findings in the presence of an LHON mtDNA mutation was described by Harding and referred to as “Harding disease” or “LHON-MS”. LHON-MS is characterized by recurrent episodes of visual loss associated with ocular pain and central nervous system demyelination along with unmatched cerebrospinal fluid oligoclonal bands. In past years, authors have extensively discussed the possibility that LHON-MS syndrome could be coincidental; an interesting point addressed in these discussions was whether and how the two diseases reciprocally influence their natural histories [6]. Upregulation of mitochondrial manganese superoxide dismutase and increased expression of inducible nitric oxide synthase within the inflammatory lesions have been described.

The 3460G > A mtDNA mutation occurs in the ND1 gene, which encodes a subunit of complex-I of the electron transport chain, NADH: ubiquinone oxidoreductase; the mutation reduces the rotenone- and ubiquinone-dependent electron transfer activity of complex without affecting the activity of proximal NADH dehydrogenase [7]. We speculate that the energy imbalance produced by this genetic defect could lead to the spinal cord manifestations seen in this patient due to the high energetic demand of the spinal cord anterior horn. In some mitochondrial diseases (e.g., MELAS), stroke-like lesions in the brain are the consequence of an energy imbalance between the demand for and the availability of ATP in neurons, astrocytes and endothelial cells [3]. Some MELAS/LHON cases have been reported, but none of these cases had spinal cord involvement. Some authors have suggested that the mitochondrial dysfunction acts as a driver of neurodegeneration both in the classic LHON presentation and in the MS-like pathology through energy deficiency, hypoxic-like tissue injury and exposure of mtDNA-encoded proteins as histocompatibility antigens [1, 8]. A progressive metabolic axonopathy was reported by Jaros [9] in a case of LHON as a result of a lifelong pathology, and no evidence of demyelination was observed in the autoptic spinal cord; it has been speculated that different mtDNA mutations predispose different neuronal types to specific susceptibility to neurodegeneration [9–11]. Spinal cord involvement during the onset of two cases of LHON mimicking neuromyelitis optica has recently been described [12, 13], emphasizing the necessity of better characterizingin its early stages. We acknowledge the possibility that our patient might have co-occurrence of the LHON mutation and inflammatory CNS pathology; indeed, it is possible that the two diseases reciprocally influence each other’s natural history. However, neither the criteria of seronegative NMO nor the MS clinical diagnostic criteria were fulfilled at one-year follow-up [14, 15]. To our knowledge, this is the first reported case of a pediatric spinal cord acute lesion that could represent the onset of neurological manifestations in a patient carrying a typical LHON mutation. Incomplete penetrance is not uncommon in LHON, and factors including additional mtDNA variants [16] and mtDNA haplogroups [17] have been proposed to influence the onset and progression of disease in patients with the LHON mutation. Direct sequencing of full mtDNA in our case ruled out a synergic role of other mtDNA mutations, and no experimental data support a role of the predicted H27 haplogroup as a genetic modifier in LHON. We cannot exclude the possibility that other factors, such as nuclear background [18, 19], might be relevant to protecting our patient from optic nerve pathology or might influence the peculiar aspects of her clinical presentation.

## Conclusions

A precise description of LHON patients presenting with extraocular neurological symptoms appears fundamental for clarifying the variability of different phenotypes and presentations and tailoring the therapeutic intervention. Early identification of new cases and monitoring of carriers beginning in childhood is important to prevent neurological and ophthalmological deterioration and preserve long-term function. Our case reports a novel pediatric clinical manifestation associated with the G3460A mtDNA mutation, broadening the clinical spectrum of this disease.

## Abbreviations

1H-MRS: Proton magnetic resonance spectorscopy
CSF: Cerebrospinal fluid
DWI: Diffusion weighted imaging
ERG: Electroretinogram
LHON: Leber’s hereditary optic neuropathy
MELAS: Mitochondrial encephalomyopathy, lactic acidosis and stroke-like episodes
MRI: Magnetic resonance imaging
MS: Multiple sclerosis
mtDNA: Mitochondrial DNA
NMO: Neuromyelitis optica.
PCR-RFLP: Polymerase chain reaction - restriction site length polymorphism
VEP: Visual evoked potential
